# Orally Administered Bifidobacterium adolescentis Diminishes Serum Glutamate Concentration in Mice

**DOI:** 10.1128/spectrum.05063-22

**Published:** 2023-06-22

**Authors:** Felix Royo, Hector Tames, Guillermo Bordanaba-Florit, Diana Cabrera, Maria Azparren-Angulo, Clara Garcia-Vallicrosa, Abelardo Margolles, Lorena Ruiz, Patricia Ruas-Madiedo, Juan M. Falcon-Perez

**Affiliations:** a Exosomes Laboratory, Center for Cooperative Research in Biosciences, Basque Research and Technology Alliance, Derio, Spain; b Centro de Investigación Biomédica en Red de Enfermedades Hepáticas Y Digestivas, Madrid, Spain; c Departamento de Microbiología y Bioquímica, Instituto de Productos Lácteos de Asturias, Consejo Superior de Investigaciones Científicas, Villaviciosa, Asturias, Spain; d Functionality and Ecology of Beneficial Microbes (MicroHealth) Group, Instituto de Investigación Sanitaria del Principado de Asturias, Oviedo, Asturias, Spain; e Metabolomics Platform, Center for Cooperative Research in Biosciences, Basque Research and Technology Alliance, Derio, Spain; f IKERBASQUE, Basque Foundation for Science, Bilbao, Spain; University of Nevada Reno

**Keywords:** *Bifidobacterium adolescentis*, glutamate, GABA, probiotic intervention, gut microbiota, gut-brain axis

## Abstract

Several studies have described the contribution of glutamate-transforming microbiota to the development of chronic ailments. For instance, the blood concentration of glutamate is higher in some patients with fibromyalgia, chronic fatigue, and pain. Taking advantage of a naturally occurring strain of *Bifidobacterium* that is able to transform glutamate in γ-aminobutyric caid (GABA), B. adolescentis IPLA60004, we designed a placebo-controlled intervention to test if the presence of this GABA-producing bifidobacteria in mice was able to impact the concentration of glutamate in the blood in comparison with the administration of other strain of the same species lacking the genes of the glutamate decarboxylase (*gad*) cluster. Animals were fed every day with 8 log CFU of bacteria in a sterilized milk vehicle for 14 days. Samples from feces and blood were collected during this period, and afterwards animals were sacrificed, tissues were taken from different organs, and the levels of different metabolites were analyzed by ultrahigh-performance liquid chromatography coupled to mass spectrometry. The results showed that both bacterial strains orally administered survived in the fecal content, and animals fed B. adolescentis IPLA60004 showed a significant reduction of their glutamate serum concentration, while a nonsignificant decrease was observed for animals fed a reference strain, B. adolescentis LGM10502. The variations observed in GABA were influenced by the gender of the animals, and no significant changes were observed in different tissues of the brain. These results suggest that orally administered GABA-producing probiotics could reduce the glutamate concentration in blood, opening a case for a clinical trial study in chronic disease patients.

**IMPORTANCE** This work presents the results of a trial using mice as a model that were fed with a bacterial strain of the species B. adolescentis, which possesses different active genes capable of degrading glutamate and converting it into GABA. Indeed, the bacterium is able to survive the passage through the gastric tract and, more importantly, the animals reduce over time the concentration of glutamate in their blood. The importance of this result lies in the fact that several chronic ailments, such as fibromyalgia, are characterized by an increase in glutamate. Our results indicate that an oral diet with this probiotic-type bacteria could reduce the concentration of glutamate and, therefore, reduce the symptoms associated with the excess of this neurotransmitter.

## INTRODUCTION

In the last few years, different studies have pointed to the relationship between the gut microbiota and the proper communication between the gut and the brain (1, 2), giving support to intervention studies manipulating the gut microbiome to modulate the pathophysiology of different disorders. For example, it has been observed that the combination of a gluten-free diet and probiotic supplementation to inhibit immune-inflammatory dysfunctions in major depressive disorders improved both psychiatric and gut barrier-associated symptoms (3). In this context, the study of bacterial strains able to influence the central nervous system (CNS) function has gained special attention (4). Among them, those bacteria able to metabolize glutamate are of special interest, because glutamate is an excitatory neurotransmitter. Besides, it is a reservoir of energy and has a role in physiological processes in the brain, for instance, in the regulation of neuronal transmission and CNS plasticity (5). Furthermore, glutamate is the major excitatory amino acid in the CNS, where elevated levels can induce acute injury (6), but simultaneously it also serves as a precursor for γ-aminobutyric caid (GABA), the primary inhibitory transmitter in the brain. An increase of the glutamate concentration in serum and cerebrospinal fluid has been observed in fibromyalgia patients (7–9). For that reason, the influence of glutamate acquired by the diet has been extensively studied, and monosodium glutamate (MSG)-related symptoms have been found to be dose related, as well as reports of a good correlation between plasma glutamate concentration and muscle pain and headache (10, 11). However, oral administration of MSG results in highly variable serum glutamate concentrations (12), and decreased consumption does not consistently reduce associated pain (13). Complementarily, it has been observed that changes in the microbiota of fibromyalgia patients point toward a decrease of genes encoding the transporter of glutamate into bacterial cytoplasm (i.e., *gadC*) and of enzymes involved in the transformation of glutamate to l-glutamine and to GABA (i.e., *gadB*) (9), offering a new therapeutic target for intervention. Indeed, addressing the positive modulation of microbiome functionality offers a great potential to provide therapeutic solutions. Specifically, the use of probiotic preparations as food supplements has become widespread in recent years, and the employment of the genus *Bifidobacterium* as probiotic has the advantage of a history of safe use (14).

Recently, an *in silico* survey pointed that within the five species of the *Bifidobacterium* genus included in the qualified presumption of safety list by the European Food Safety Authority (EFSA) (15), only B. adolescentis genomes showed the highest prevalence of *gad* genes, suggesting this bifidobacterial taxon as a model GABA producer within this genus (16). In addition, GABA production from culture medium supplemented with MSG was demonstrated by several strains of these species having different capabilities of substrate bioconversion. In the present study, we attempted to modulate the levels of glutamate in mice by oral administration of B. adolescentis IPLA60004 (also named 2BCM2), a strain with one of the highest (about 68%) MSG bioconversion abilities among those listed in our previous work (16).

## RESULTS

### Growth and viability of *Bifidobacterium* spp. in fecal samples.

**FIG 1:**
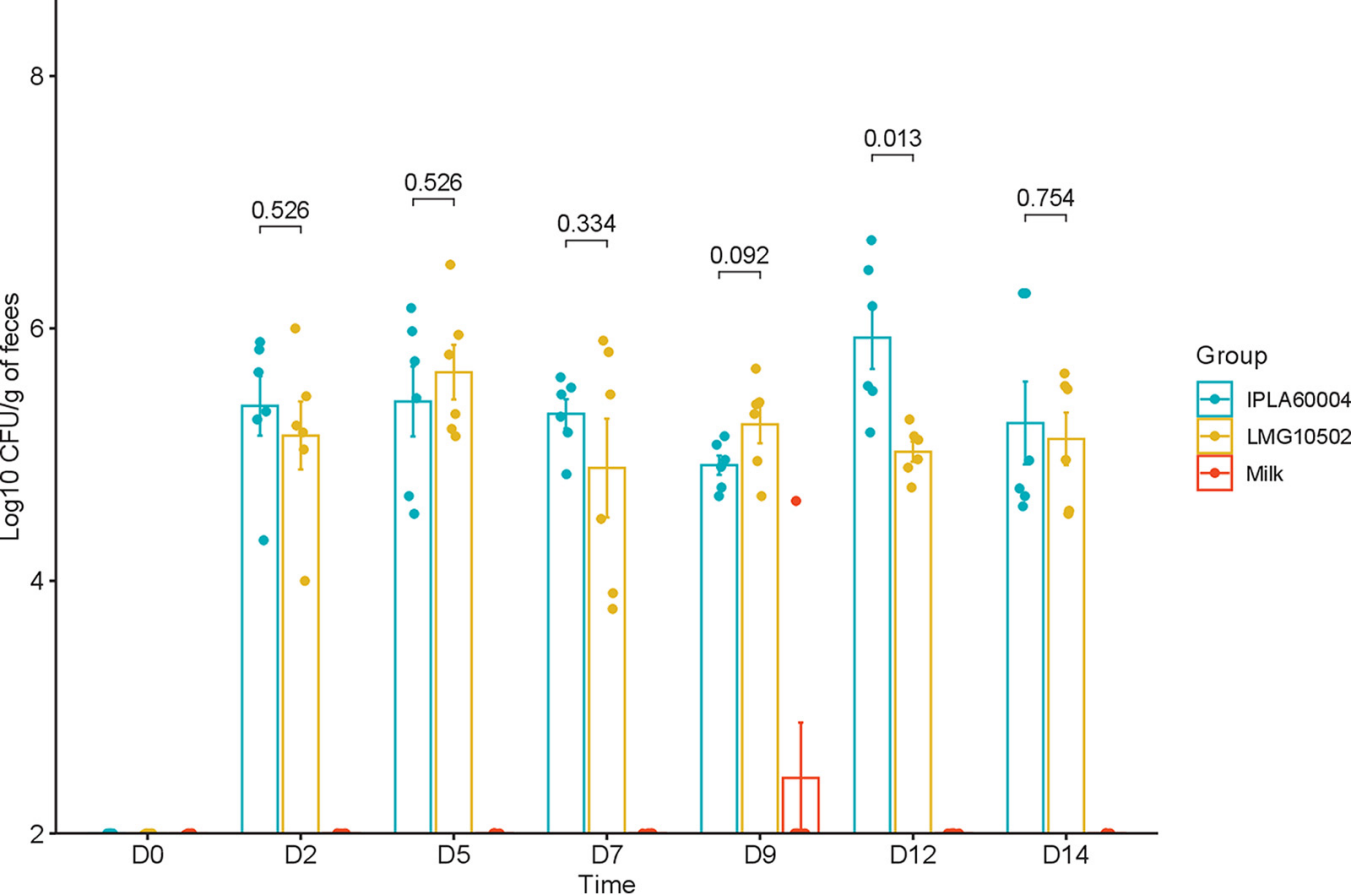
Quantification of *Bifidobacterium* recovered from culturing fecal samples from mice. The number of bacteria was expressed as the log_10_ CFU per gram of feces. For each sampling day, 6 fecal samples (3 from males and 3 from females) were collected for each of the three treatment groups. The detection limit of the dilutions plated was 2 log CFU (200 CFU/g of feces).

Culture of fecal samples in a selective medium revealed the presence of viable *Bifidobacterium* in the stool of animals fed any of both bifidobacterial strains at levels equal or higher than 5 logs CFU/g (Fig. 1). This meant that the percentage of bifidobacteria that reached the colon alive was, at the least, around 0.1%. The viable bifidobacterial counts were below the detection limit (2 logs) in animals fed the vehicle, as well as in the basal samples before the intervention (0 days), when we analyzed animals housed in the six cages used in this study (Fig. 1).

### Changes of metabolites in serum.

**FIG 2:**
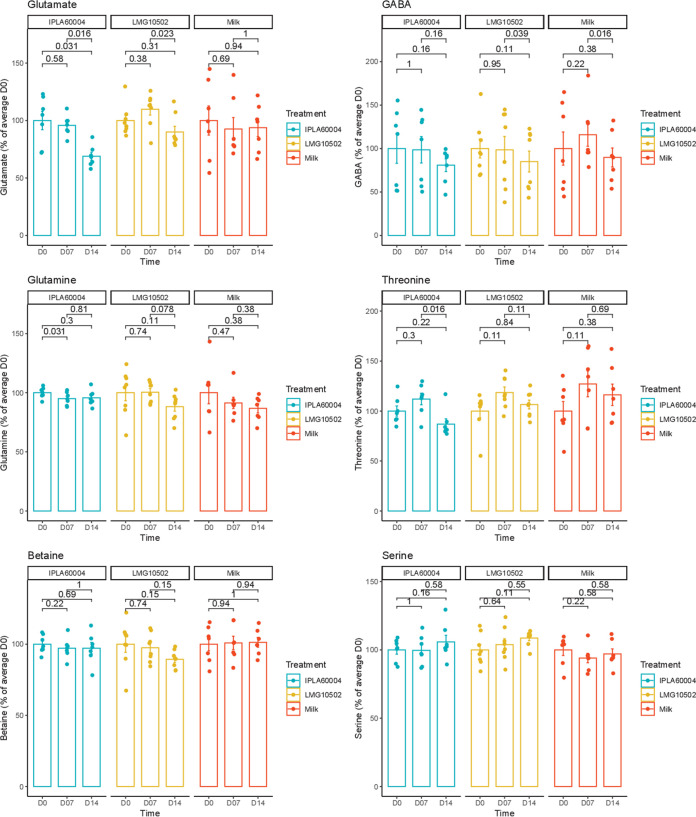
Changes along time of a selected panel of metabolites, comparing the animals that received each bifidobacterial strain or the vehicle (milk). Bars represent averages and standard errors of the means (SEM) (*n* = 8). Relative units of the *y* axes are the relative percentage at D0 of each respective group, and the *post hoc* analysis used a paired Wilcoxon test. Raw data values and their statistical analysis are presented in Table S1 in the supplemental material.

**FIG 3:**
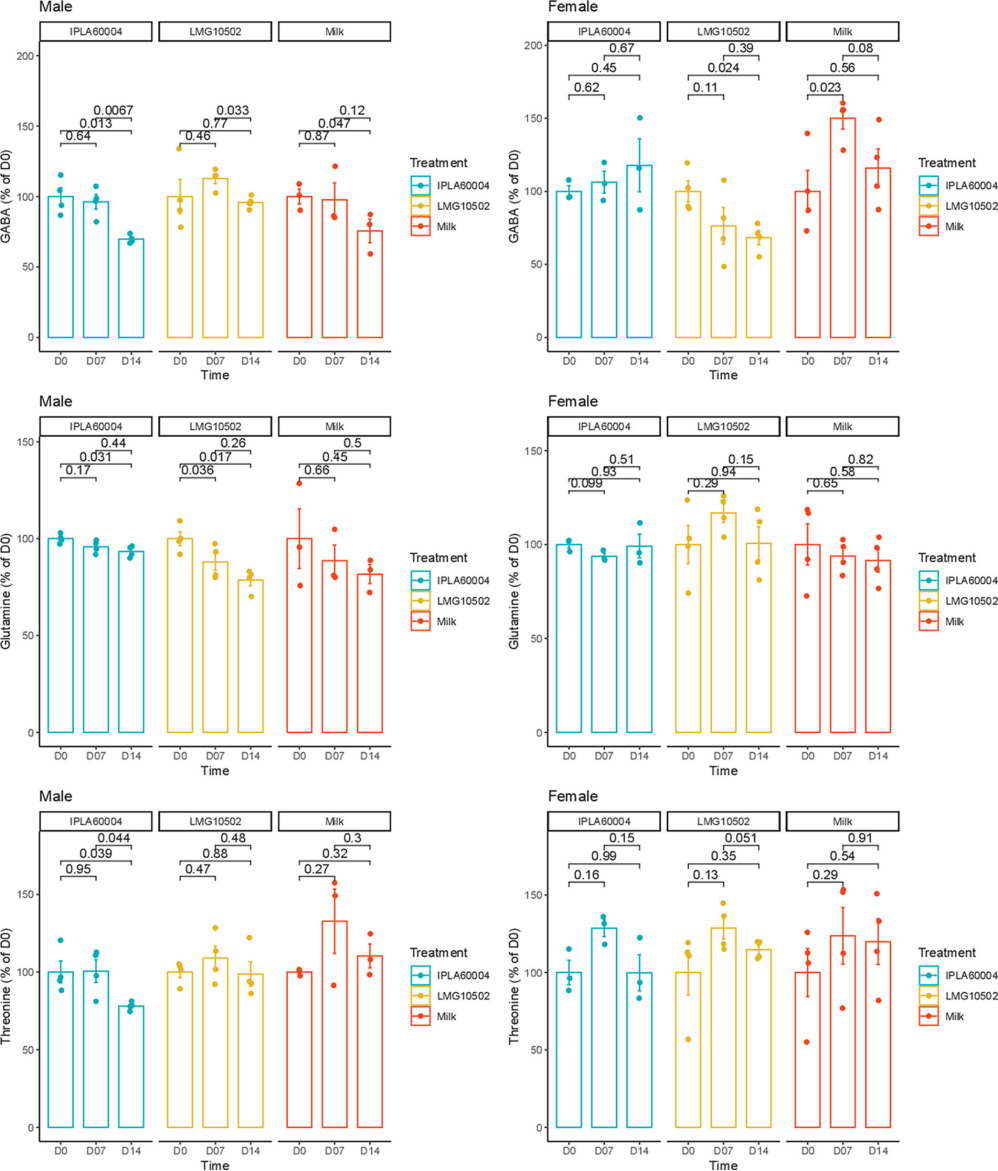
Changes along time of those metabolites that showed differences by gender. Bars represent averages and SEM (*n* = 4). Relative units of the *y* axes are relative percentage with respect to the average at D0 of each respective group, and the *post hoc* analysis used a paired *t* test. ANOVA results as well as raw values are presented in Table S1.

Blood sampling was performed prior to (D0), during (D7), and at the end (D14) of the experimental time. In Fig. 2, it can be observed that the evolution along the time for each metabolite analyzed relative to D0, which included glutamate and GABA, directly affected by bacterial metabolism, glutamine was involved in the metabolic route of glutamate and GABA. Another two amino acids (threonine and serine), as well as the amino acid-derived metabolite betaine, were analyzed as controls of the individual changes of the metabolome profile along time. The complete data, including the averages and standard deviations of adjusted intensity areas, as well as the *P* values obtained after a repeated analysis of variance (ANOVA) for gender, time, and treatment, is presented in Table S1 in the supplemental material. Among the metabolites of interest for this study, only threonine and glutamate gave a significant result for both factors, time and treatment, simultaneously, but in the case of threonine, there was also a significant effect due to gender (Table S1). There was a statistically significant reduction of glutamate during the experiment for the animals treated with B. adolescentis IPLA60004, comparing D14 versus D0 and D7 with D0, while the decrease observed for the animals fed B. adolescentis LMG10502 was not significant for either intervention time with respect to the basal (D0) level. GABA, glutamine, and threonine were also strongly influenced by gender (Table S1), which was the reason why we also analyzed separately the data by gender (Fig. 3). As shown in this Fig. 3, we observed differences in males regarding a decrease at D14 compared to D0 for GABA, glutamine, and threonine in those animals treated with B. adolescentis IPLA60004. We also observed a decrease of glutamine for the males that received B. adolescentis LMG10502. In the case of females, the results seem to be steadier for all the metabolites, and we only observed a decrease of GABA for females treated with B. adolescentis LMG10502. We should also mention that, even on day 0, we identified variations in certain metabolites across groups when we performed an ANOVA without taking into account the animal or time factors (Table S2). Less ambiguous findings included the reduced choline and glycerophosphocholine values in the vehicle group on days 7 and 14. However, the concentrations for those metabolites in the vehicle group were likewise lower on D0 (though not significantly lower), as shown in Table S1 (the reason why these results were not significant in the repeated-measures analysis shown in Table S1).

### Metabolic changes in the cortex and hypothalamus.

**FIG 4:**
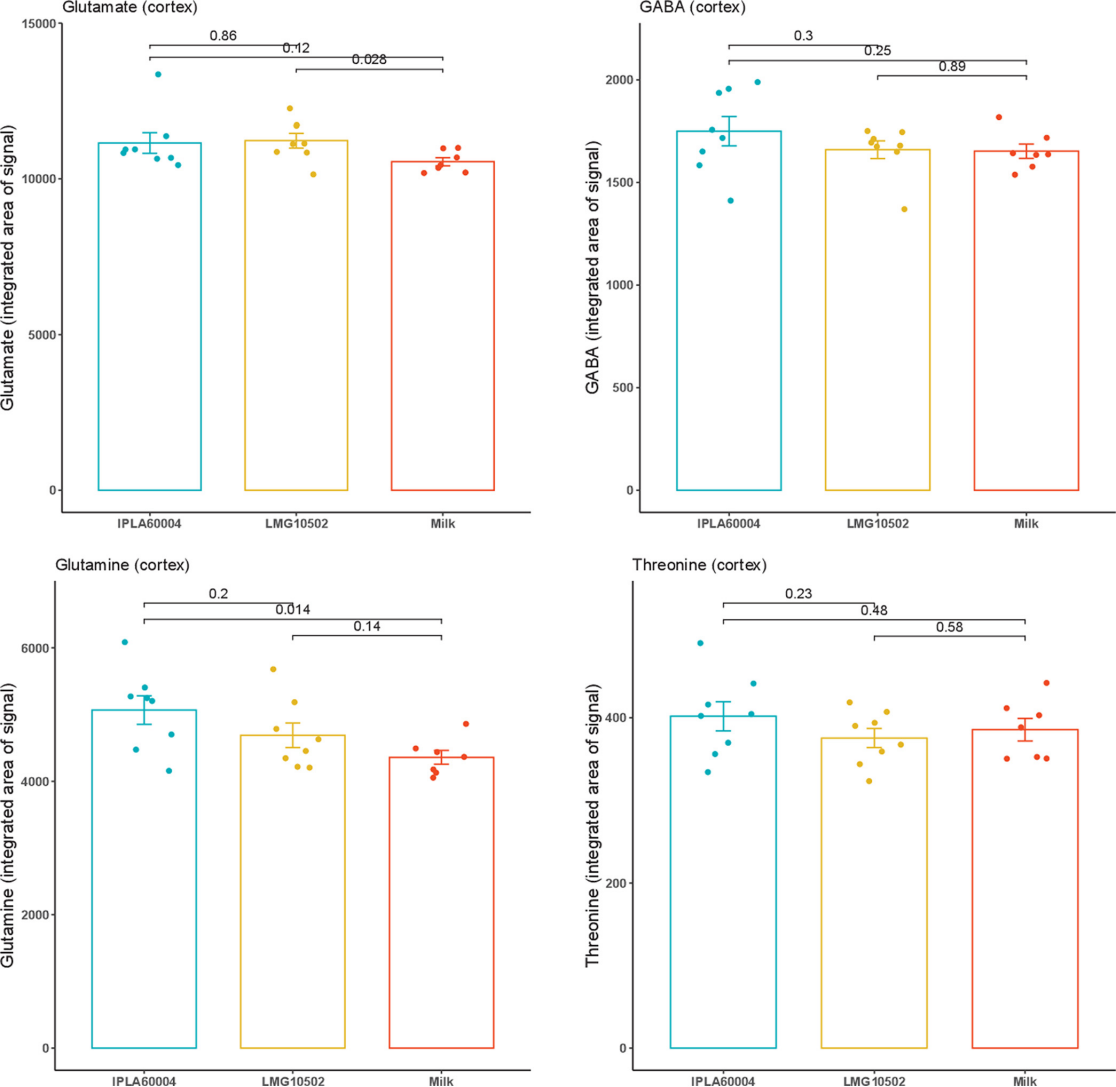
Comparison of the changes of metabolites in the cortex upon treatments. Bars represent averages and SEM (*n* = 8) of the integrated areas of the detection signals. The *post hoc* analysis used a *t* test, and ANOVA results are presented in Table S3.

**FIG 5:**
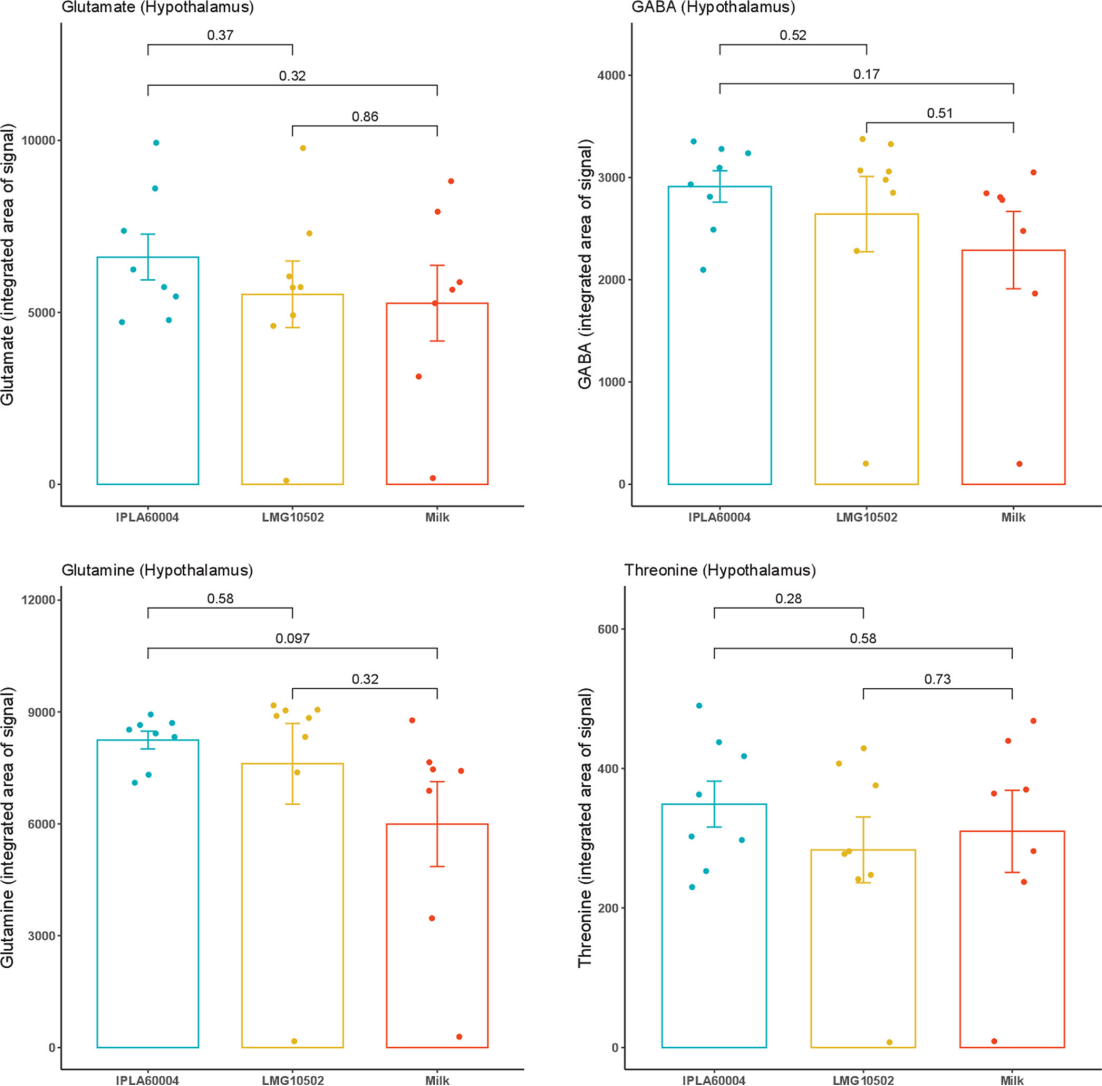
Comparison of the changes of metabolites in the hypothalamus upon treatments. Bars represent averages and SEM (*n* = 8) of the integrated areas of the detection signals. The *post hoc* analysis used a *t* test, and ANOVA results are presented in Table S4.

To follow the changes observed in serum, we also measured the levels of different metabolites in two different areas of the brain. In this case, the samples were collected at the end of the experiment (D14), from dissected areas of cortex and hypothalamus. Figure 4 shows comparisons between the different treatments for the levels of measured metabolites in the cortex, and Fig. 5 shows the levels in the hypothalamus. The averages, standard deviations (SD), and *P* values calculated by two-way ANOVA are provided in Tables S3 and S4. We observed that values for animals treated only with vehicle were slightly lower for glutamine at the end of experiment in the case of the cortex, and no significant differences were detected in the case of hypothalamus. There were not statistical differences between the groups fed both bifidobacterial strains.

**FIG 6:**
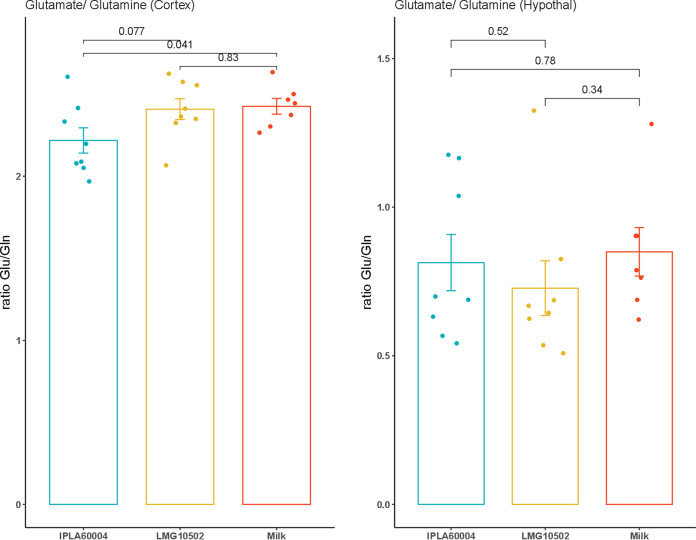
Comparison of the ratios between glutamate and glutamine in the cortex and hypothalamus in the animals of the different groups at the end of experiment. The values represent the ratios between the integrated areas of the detection signals, and *post hoc* analysis used a *t* test (*n* = 8).

In addition, we calculated the differences among groups in the glutamate/glutamine ratios, and the results are presented in Fig. 6. We observed that the ratio in the cortex was significantly lower for animals treated with the strain B. adolescentis IPLA60004 than for the animals treated with B. adolescentis LMG10502 or the vehicle, while no differences were observed in the hypothalamus.

## DISCUSSION

The present study confirms that following oral administration of both B. adolescentis strains, high rates of survival were obtained, as the bacterium was able to overcome the passage through the gastrointestinal tract, as previously observed in a rat model (16). This number of viable bacteria, more than 10^5^ CFU per g of feces, suggests the potential use of B. adolescentis in probiotic interventions toward modulating the level of GABA precursors in the gut, since the bacteria must be metabolically active within the gut to conduct the transformation of glutamate and glutamine into GABA. For this particular target of action, having sufficient survival after the gastrointestinal transit, as well as maintaining viability during its inclusion in the delivery vehicle (milk in this case), are crucial for selection of probiotic strains before being applied for specific target populations (17).

The effect of the *Bifidobacterium* strains administered resulted in changes in the concentrations of glutamate in serum. After 2 weeks of bacteria administration, without diet changes among groups, there was a significant decrease of the serum concentration for the animals receiving B. adolescentis IPLA60004. In the case of animals treated with B. adolescentis LMG10502, we observed a decrease between day 7 and 14, but the reduction between days 0 and 14 did not reach statistical significance, and so we could not claim a decrease during the duration of the experiment. Since a high concentration of glutamate in plasma has been found in patients with muscular myalgia (18), the possibility of reducing glutamate concentrations in blood with a probiotic intervention could be considered a promising result. In our study, we dissected separately the frontal cortex and the hypothalamus, and the concentrations of glutamate in those tissues did not reflect the differences observed upon treatments in blood. On the other hand, there was a decrease of the glutamate/glutamine ratio. Since we have observed that individual variation of glutamate concentration in serum occurs, the ratio glutamate to glutamine may be more indicative of the balance of amino acids at the time of sacrifice. High levels of glutamate and glutamine in the posterior gyrus of the cortex have been associated with fibromyalgia (19) and migraine (20). In addition, different studies have described that a concentration gradient established between the endothelial cell and the blood facilitates glutamate transport from cerebrospinal fluid and brain tissue to the peripheral blood by facilitated diffusion (21). The brain-to-blood glutamate efflux is a rapid and naturally occurring event (22). The impact of dietary intake on daily variations in blood glutamate concentrations and subsequent modulation of efflux is not yet fully understood. However, it has been suggested that dietary intake and metabolism may play a role in this process (23). Low-glutamate diets have been associated with positive effects, potentially due to this modulation (24). Notably, changes in blood glutamate levels were not accompanied by parallel increases in GABA levels, highlighting the specificity of homeostatic mechanisms for each metabolite. In this regard, it has been observed that medium doses of orally administered GABA increase GABA levels in blood, while high doses of GABA decrease the circulating concentration and increased succinic semialdehyde dehydrogenase activity in the liver (25).

Our results suggest that the presence of specific B. adolescentis strains in the gut could be advisable to patients with maladies associated with high levels of glutamate, as a way of improving and complementing the effects of a low-glutamate diet. This specific diet can effectively reduce overall symptoms such as pain and fatigue, but those results upon challenge suggest that other aspects of the diet, or underlying differences within the population, are involved (24).

Although previous studies using B. adolescentis in rats found a slight, nonsignificant increase of fecal GABA (16), we did not observe an increase of GABA in serum or brain tissues. However, we observed significant differences for animal gender for GABA concentration in serum, a difference that has not been pointed out in human studies of GABA in serum (26, 27) and therefore could be intrinsic to the animal model employed. There are also daily variations, as reflected in significant differences in the vehicle group between days 7 and 14. Indeed, the serum levels of some metabolites were strongly influenced by gender, with a tendency to decrease in the last days of the experiments for males, as observed for glutamine and threonine. One possibility relies on differences in the metabolism between genders, which also has been observed in the changes of serum metabolites after fecal replacement associated with stress-induced hyperalgesia in mice (28). Moreover, early stress alters microbiota differently depending on gender, making the relationships between microbiota intervention and the outcome quite dependent on age and gender (29). Finally, we observed other variations between vehicle treatment and probiotic treatment even before the start of the experiment (D0), showing intrinsic individual variations. For these reasons, the observed effect over glutamate of the probiotic intervention, as it was reduced in both males and females only after treatment with B. adolescentis IPLA60004, reinforces the idea of its possible suitability for clinical trials.

Nevertheless, the importance of glutamine, GABA, and glutamate concentrations in different brain tissues are important in chronic pain pathogenesis (20), and while high levels of glutamate are associated with pain, the decrease of glutamate is associated with cognitive disabilities in patients with fibromyalgia (30) and depression in elderly people. Therefore, to regulate the concentration of these metabolites could be key in treatment of some chronic pain conditions. The present study reinforces the potential of using probiotics and diet intervention to modulate glutamate concentration.

## MATERIALS AND METHODS

### Bacterial strains and growth conditions.

Two B. adolescentis strains were used in this work. The strain IPLA60004 belongs to IPLA-CSIC culture collection, and it was previously isolated from a human colonic biopsy specimen; it has a high capability to convert MSG into GABA. The strain LMG10502 was purchased from the Belgium culture collection (BCCM/LMG Bacteria Collection, Ghent, Belgium) and it lacks *gad* genes. Strains were grown in MRSc medium (MRS [Biokar Diagnostic, Beauvais, France] supplemented with 0.25% l-cysteine hydrochloride monohydrate [Sigma-Merck, Darmstadt, Germany]). Stocks stored at –80°C were grown in agar-MRSc in anaerobic jars with Anaerocult A (Merck) at 37°C for 2 days. Isolated colonies were inoculated in 50 mL of MRSc broth and incubated under the same conditions for 20 ± 1 h to obtain the bifidobacterial cultures.

### Probiotic preparation for oral administration.

Bacterial cultures were washed once with sterile phosphate-buffered saline (PBS) solution and resuspended in 5 mL (10× concentration) of sterilized milk prepared as follows. Skimmed milk powder (BD-Difco, Merck) was resuspended (11%) in water, well homogenized, and sterilized by autoclaving (121°C, 15 min). The bifidobacterial-milk suspensions were prepared daily during the intervention period, and viable bacteria were enumerated by plating serial dilutions (made in Ringer ¼, Merck) of the suspensions on agar-MRSc. After incubation under standard conditions, the log CFU per milliliter levels of the bifidobacterial suspensions were 9.20 ± 0.36 and 9.23 ± 0.32 for strains LMG10502 and IPLA60004, respectively. Finally, each animal was orally administered daily with 100 μL of bifidobacterial suspensions or vehicle (sterilized milk); thus, groups administered with probiotic strains received a dose of about 8.0 log CFU per day (100, 000,000 CFU administered in 100 μL).

### Animals and *in vivo* study design.

All animal experimentation was conducted in accordance with Spanish guidelines for the care and use of laboratory animals, and protocols were approved by the CIC bioGUNE Institute and the regional Basque Country Ethical Committee (ref. P-CBG-CBBA-1521).

C3H/HeJ mice, commonly called C3H, were purchased from the Jackson Laboratory (Bar Harbor, Ma, USA) at the age of 9 weeks. Animals were kept under standard conditions of the animal facility for 1 week before the beginning of the experiment, in an environmentally controlled room at 22°C on a 12-h light, 12-h dark cycle and provided with standard diet and water *ad libitum*. In total, 24 animals were caged in boxes of 4 animals, forming 3 groups of four males and four females for each treatment. Animals of different sexes or different treatments were not caged together. All the animals of the cage were fed by oral gavage with sterilized milk (vehicle), the probiotic B. adolescentis IPLA60004 strain, or reference B. adolescentis LMG10502 strain, depending on the group. On days 0 (initial), 2, 5, 7, 9, 12, and 14 (final), animals were weighed, and fecal samples were obtained to monitor viable counts of *Bifidobacterium* spp. Blood samples were taken at basal time (D0) and after 7 days (D7) of intervention and stored under frozen conditions. To avoid confounder effect, a different order of the sampling and exchange of operators between groups were used for each sample time. Finally, at day 14 animals were sacrificed, and blood, hypothalamus, and cortex tissues were taken and deep-frozen immediately for further metabolomic analysis.

### Quantification of fecal *Bifidobacterium*.

The quantification of viable bifidobacteria in fecal samples of mice was performed as previously described (31). In short, three feces were randomly collected from each of the six cages housing the animals as previously described, at different intervention days: 0 (initial), 2, 5, 7, 9, 12, and 14 (final). The same day, feces were weighed and mixed with PBS (final concentration, 10 mg/mL) to be properly homogenized using a Stomacher under stirring for 2 min. Finally, serial dilutions from the fecal homogenates were made in Ringer ¼, which were plated on the surface of agar-propionate-transgalctosylated oligiosaccharide agar medium (agar-proponionate-TOS medium) (Millipore, Merck), supplemented with lithium-mupirocin as recommended by the manufacturer. This medium allows the selective counting of viable bifidobacteria (as CFU per gram of feces) after incubation at 37°C under anaerobic conditions for at least 2 days.

### Serum sample preparation.

To 40-µL aliquots of serum, 40 µL of water containing 0.15% formic acid was added. Subsequently, proteins were precipitated by addition of 120 µL of acetonitrile. In order to get optimum extraction, after addition of acetonitrile the samples were sonicated for 10 min at 4°C and agitated at 1,400 rpm for 30 min at 4°C. Next, the samples were centrifuged at 14,000 rpm for 30 min at 4°C. The supernatant was transferred to a fresh vial for ultrahigh-performance liquid chromatography–mass spectrometry (UHPLC-MS) analysis.

### Brain tissue sample preparation.

Tissues (cortex and hypothalamus samples between 10 mg and 100 mg) were homogenized in 500 µL of ice-cold extraction liquid with a tissue homogenizer (FastPrep) in a 40-s cycle at 6,000 rpm. The extraction liquid consisted of a mixture of ice-cold methanol-water (50/50 [vol/vol]). Subsequently, 400 µL of the homogenate plus 400 µL of chloroform was transferred to a new tube and shaken at 1,400 rpm for 60 min at 4°C. Next, the samples were centrifuged for 30 min at 13,000 rpm at 4°C. The organic phase was separated from the aqueous phase. From the aqueous phase, 250 µL was transferred to a fresh tube and placed at –80°C for 20 min. The chilled supernatants were evaporated with a Speedvac in approximately 2 h. The resulting pellets were resuspended in 150 µL water-acetonitrile (40/60 [vol/vol]).

### UHPLC-MS analysis.

Extracts from brain tissues and serum samples were measured with an ultraperformance liquid chromatographic system (Acquity, Waters Inc., Manchester, United Kingdom) coupled to a time-of-flight mass spectrometer (Synapt G2, Waters Inc.). A 2.1- by 100-mm, 1.7-µm ethylene-bridged hybrid amide column (Waters Inc.), with thermostat at 40°C, was used to separate the analytes before entering into the mass spectrometer. Mobile phase solvent A (aqueous phase) consisted of 99.5% water, 0.5% formic acid, and 20 mM ammonium formate, while solvent B (organic phase) consisted of 29.5% water, 70% acetonitrile, 0.5% formic acid, and 1 mM ammonium formate.

In order to obtain a good separation of the analytes, the following gradient was used: from 5% A to 50% A in 2.4 min in curved gradient (number 8, as defined by Waters), from 50% A to 99.9% A in 0.2 min, constant at 99.9% A for 1.2 min, and back to 5% A in 0.2 min. The flow rate was 0.250 mL/min and the injection volume was 2 µL. All samples were injected randomly, and after every 6 injections a quality control sample was injected.

The MS was operated in positive electrospray ionization in full scan mode. The cone voltage was 25 V and capillary voltage was 250 V. Source temperature was set to 120°C and capillary temperature to 450°C. The flow of the cone and desolvation (both nitrogen gas) was set to 5 liters/h and 600 liters/h, respectively. A 2-ng/mL leucine-enkephalin solution in water-acetonitrile-formic acid (49.9/50/0.1 [vol/vol/vol]) was infused at 10 µL/min and used for a lock mass, which was measured each 36 s for 0.5 s. Spectral peaks were automatically corrected for deviations in the lock mass. To optimize fragmentation, trap collision energy was set to 6 V.

Extracted ion traces for relevant analytes were obtained in a 20-mDa window in their expected *m/z* channels. These traces were subsequently smoothed, and peak areas were integrated with TargetLynx software (Waters).

These calculated raw signals were adjusted by median fold change (MFC) adjustment. This is a robust adjustment factor for global variations in signal due to, e.g., differences in tissue amounts, signal drift, or evaporation. The MFC is based on the total amount of detected mass spectrometric features (unique retention time/mass pairs). The calculations and performance of the MFC adjustment factors have been described elsewhere (32, 33).

### Statistical analysis.

The number of animals was decided by a power *t* test, to be able to detect a difference between averages of 1.5× the SD, with a power of 0.8 and significance of 0.05 (two side). To compare the treatment effect in each animal (experimental unit) along the experimental duration, taking into account gender, a repeated measures model taking time as the within variable and gender and treatment as in-between variables, was fed to the function anova_test of the package rstatix (https://rpkgs.datanovia.com/rstatix).

### Data availability statement.

We confirm that the data supporting the findings of this study are available within the article and its supplemental materials. The raw data generated from the UHPLC-MS analysis are also available from the corresponding author, J.M.F.-P., on request.
