# Supplementary material for: Orally Administered Bifidobacterium adolescentis Diminishes Serum Glutamate Concentration in Mice
Source: Microbiol Spectr. 2023 Jun 22;11(4):e05063-22. doi: 10.1128/spectrum.05063-22 (PMC10433951; doi:10.1128/spectrum.05063-22)
Supplement: Supplemental file 1 — Table S1. Download spectrum.05063-22-s0001.pdf, PDF file, 0.04 MB [file spectrum.05063-22-s0001.pdf]

Supplementary Table I. Average +/- SD of the integrate signal for each metabolite in the serum

Metabolites	ANOVA			D0							
				B. adolescentis LMG10502		B. adolescentis IPLA60004		Vehicle		B. adolescentis LMG10502	
Gender	Gender	Treatment	Time	Female	Male	Female	Male	Female	Male	Female	Male
Betaine	0.864	0.579	0.389	20917.88+/-4880.4	21304.44+/-1753.3	21131.6+/-1477.91	21057.75+/-1480.11	19149.22+/-2841.1	21227.93+/-2102.04	21645.81+/-2299.37	19505.99+/-1399.75
Choline	0.029	1.61E-07	0.055	13087.56+/-2319.39	12237.66+/-400.51	13537.98+/-1225.29	11660.63+/-829.43	10628.81+/-1069.43	10849.4+/-989.76	14094.49+/-1997.22	12579.19+/-629.53
GABA	1.86E-10	0.294	5.08E-04	6.01+/-0.87	9.3+/-2.23	4.54+/-0.31	11.41+/-1.42	4.56+/-1.31	11.89+/-1.53	4.59+/-1.5	10.49+/-0.67
Glutamate	3.70E-01	3.96E-05	1.20E-02	141.39+/-26.59	132.69+/-4.92	140.51+/-36.4	146.92+/-30.32	93.72+/-39.21	122.79+/-29.63	150.7+/-11.76	150.15+/-27.67
Glutamine	8.53E-04	6.03E-04	0.066	1704.58+/-346.46	2243.4+/-159.66	2114.16+/-69.67	2269.11+/-54.05	2132.71+/-466.34	2674.2+/-584.88	1989.9+/-168.4	1974.22+/-193.14
GPC	1.53E-04	1.37E-05	7.40E-01	39297.06+/-13177.42	31375.04+/-2796	41003.4+/-2395.31	29180.85+/-3615.89	23855.97+/-7949.24	19510.02+/-2549.46	38790.45+/-6700.67	30877.15+/-1752.04
Serine	0.135	0.591	0.192	46.39+/-6.99	43.43+/-2.53	47.86+/-1.22	44.43+/-4.57	45.11+/-6.55	50.31+/-2.76	50.66+/-4.12	42.59+/-2.96
Spermine	0.606	0.071	0.197	182.13+/-52.39	157.36+/-73.39	224.8+/-126.7	191.48+/-101.99	189.43+/-161.27	240.45+/-99.55	155.02+/-63.31	114.99+/-32.07
Threonine	0.025	0.004	0.006	393.24+/-114.06	416.38+/-30.51	428.47+/-58.84	464.29+/-65.51	353.91+/-109.87	341.27+/-89.22	506.13+/-56.1	453.72+/-65.15

D7				D14					
B. adolescentis IPLA60004		Vehicle		B. adolescentis LMG10502		B. adolescentis IPLA60004		Vehicle	
Female	Male	Female	Male	Female	Male	Female	Male	Female	Male
19880.86+/-1312.5	20902.48+/-1549.7	19512.93+/-2800.65	20580.47+/-2309.26	18752.12+/-1386.8	18966.99+/-1457.66	19831.84+/-3027.38	20930.45+/-794.74	20938.9+/-1523.91	18870.64+/-1521.1
13069.2+/-962.62	10830.1+/-1369.91	8443+/-1275.3	8332.74+/-398.05	12746.67+/-619.39	12481.21+/-612.23	11407.88+/-478.48	12329.75+/-1131.91	9366.91+/-700.82	8889.08+/-928.11
4.88+/-0.49	10.97+/-1.19	6.84+/-0.67	10.99+/-2.32	4.11+/-0.57	8.91+/-0.4	5.61+/-1.27	7.96+/-0.34	5.29+/-1.21	8.51+/-1.64
134.32+/-12.31	136.84+/-17.14	88.88+/-22.78	103.41+/-34.68	125.74+/-22.94	121.13+/-17.6	100.35+/-16.86	96.98+/-8.02	86.89+/-18.33	108.68+/-20.14
1954.19+/-74.58	2174.79+/-73.57	2001.99+/-182.69	2304.43+/-363.86	1715.93+/-301.59	1764.8+/-130.77	2031.56+/-230.54	2118.76+/-65.96	1949.97+/-259.14	2122.69+/-221.48
41109.51+/-3857.81	27829.15+/-4805.82	24360.83+/-7630.99	22749.48+/-3439.37	39138.81+/-3142.91	30367.53+/-1754.01	32854.27+/-5977.77	28069.46+/-772.49	26863.86+/-6541.94	20863.76+/-2389.15
48.61+/-8.39	46.38+/-5.7	45.06+/-4.94	42.49+/-3.47	49.01+/-1.49	48.56+/-3.41	49.48+/-6.76	46.61+/-4.74	46.24+/-6.26	44.26+/-0.55
106.57+/-32.17	177.55+/-95.5	163.02+/-51.36	172.14+/-47.32	129.65+/-40.04	101.28+/-27.35	136.46+/-38.84	140.48+/-68.83	186.45+/-133.23	312.69+/-109.08
546.55+/-33.75	466.92+/-67.66	437.86+/-129.19	393.77+/-106.7	450.98+/-23.16	411.05+/-66.51	408.53+/-80.65	362.62+/-12.64	424.46+/-104.54	327.44+/-39.44
