# Supplementary material for: Orally Administered Bifidobacterium adolescentis Diminishes Serum Glutamate Concentration in Mice
Source: Microbiol Spectr. 2023 Jun 22;11(4):e05063-22. doi: 10.1128/spectrum.05063-22 (PMC10433951; doi:10.1128/spectrum.05063-22)
Supplement: Supplemental file 2 — Table S2. Download spectrum.05063-22-s0002.pdf, PDF file, 0.04 MB [file spectrum.05063-22-s0002.pdf]

Supplemental Table 2. Anova and post-hoc Tukeys test for each Time and Sex of differences on serum

The groups are denotted as V (Vehicle), A (LGM10502), B(IPLA60004)

	ANOVA D0					
	Female			Male		
Metabolites	pval	Contrast		pval	Contrast	
Betaine	0.711			0.98		
Choline	0.093			0.089		
GABA	0.117			0.142		
GPC	0.07			7.98E-04	V-A	V-B
Glutamate	0.15			0.416		
Glutamine	0.232			0.206		
Serine	0.834			0.038	V-A	
Spermine	0.892			0.47		
Threonine	0.641			0.075		

	ANOVA D7					
	Female			Male		
Metabolites	pval	Contrast		pval	Contrast	
Betaine	0.389			0.521		
Choline	9.50E-04	V-A	V-B	0.001	V-A	V-B
GABA	0.0208	V-A	V-B	0.864		
GPC	8.83E-03	V-A	V-B	0.051		
Glutamate	1.29E-03	V-A	V-B	0.122		
Glutamine	0.897			0.198		
Serine	0.455			0.406		
Spermine	0.283			0.389		
Threonine	0.232			0.477		

	ANOVA D14					
	Female			Male		
Metabolites	pval	Contrast		pval	Contrast	
Betaine	0.383			0.096		
Choline	8.64E-05	V-A-B		0.001	V-A	V-B
GABA	0.168			0.361		
GPC	0.0328	V-A		2.15E-04	V-A	V-B
Glutamate	0.055			0.151		
Glutamine	0.269			0.0126	V-A	A-B
Serine	0.667			0.341		
Spermine	0.593			0.012	V-A	V-B
Threonine	0.743			0.11		
