# Supplementary material for: Orally Administered Bifidobacterium adolescentis Diminishes Serum Glutamate Concentration in Mice
Source: Microbiol Spectr. 2023 Jun 22;11(4):e05063-22. doi: 10.1128/spectrum.05063-22 (PMC10433951; doi:10.1128/spectrum.05063-22)
Supplement: Supplemental file 3 — Table S3. Download spectrum.05063-22-s0003.pdf, PDF file, 0.03 MB [file spectrum.05063-22-s0003.pdf]

Supplementary Table 3. Average +/- SD of the integrate signal for each metabolite in the cortex

Metabolites	ANOVA		B. adolescentis LMG10502		B. adolescentis IPLA60004		Vehicle	
	Gender	Treatment	Female	Male	Female	Male	Female	Male
Betaine	0.008	0.89	3339.61+/-530.86	2727.84+/-76.2	3217.87+/-475.73	2774+/-293.64	3089.41+/-314.25	2799.32+/-152.06
Choline	0.256	0.058	18664.92+/-977.69	19192.26+/-1313.8	20551.92+/-3018.98	21683.75+/-2828.35	18156.64+/-1147.87	19423.96+/-1239.17
GABA	0.084	0.396	1627.28+/-173.72	1693+/-42.91	1677.48+/-76.34	1824.02+/-275.33	1598.37+/-49.33	1724.92+/-90.95
Glutamate	0.615	0.134	10955.81+/-646.5	11493.3+/-633.21	11626.08+/-1176.88	10677.78+/-207.39	10555.87+/-347.5	10539.93+/-412.52
Glutamine	0.323	0.046	4409.52+/-178.76	4966.81+/-620.67	5051.56+/-823.89	5081.46+/-412.69	4335.74+/-195.03	4387.83+/-406.16
GPC	0.103	0.296	97580.79+/-3511.5	100283.51+/-4101.65	100970.2+/-16614.62	84086.04+/-9918.09	95358.38+/-5482.64	90525.78+/-1665.99
Spermidine	0.541	0.676	455.31+/-58.7	442.3+/-103.19	465.31+/-60.61	477.3+/-73.92	453.26+/-30.69	515.32+/-123.25
Spermine	0.046	0.023	3965.32+/-548.75	4140.79+/-610.81	4453.22+/-749.75	5933.07+/-1463.85	4887.35+/-222.71	5536.79+/-989.89
Threonine	0.282	0.42	373.8+/-41.54	377.2+/-27.53	374.29+/-34.8	429.48+/-50.37	387.14+/-44.09	383.67+/-30.84
