# Supplementary material for: Orally Administered Bifidobacterium adolescentis Diminishes Serum Glutamate Concentration in Mice
Source: Microbiol Spectr. 2023 Jun 22;11(4):e05063-22. doi: 10.1128/spectrum.05063-22 (PMC10433951; doi:10.1128/spectrum.05063-22)
Supplement: Supplemental file 4 — Table S4. Download spectrum.05063-22-s0004.pdf, PDF file, 0.03 MB [file spectrum.05063-22-s0004.pdf]

Supplementary Table 4. Average +/- SD of the integrate signal for each metabolite in the hypothalamus

Metabolites	ANOVA		B. adolescentis LMG10502		B. adolescentis IPLA60004		Vehicle	
	Gender	Treatment	Female	Male	Female	Male	Female	Male
Betaine	0.121	0.121	6985.8+/-1023.01	4643.34+/-2467.08	6861.07+/-576	6600.15+/-443.32	5247.29+/-2574.33	4458.86+/-1307.37
Choline	0.918	0.31	44365.99+/-2613.93	29984.7+/-20020.68	31043.52+/-13373.87	39053.22+/-7346	23084.79+/-15328.78	31131.73+/-8135.33
GABA	0.917	0.429	3153.04+/-245.6	2132.77+/-1333.8	2717.77+/-542.72	3105.39+/-212	2066.13+/-1253.52	2587.05+/-633.52
Glutamate	0.478	0.575	5915.93+/-1106.54	5131.9+/-3967.75	6938.19+/-2274.81	6271.6+/-1665.93	5699.06+/-3879.54	4685.52+/-1358.56
Glutamine	0.559	0.259	8817.46+/-339.18	6404.82+/-4231.41	8164.67+/-724.92	8330.38+/-708.21	5852.63+/-3792.69	6179.31+/-2351.05
GPC	0.578	0.166	135808.49+/-23303.17	107366.76+/-70784.68	165391.7+/-16806.78	161841.58+/-5673.9	120899.94+/-81760.22	117892.17+/-40823.56
Spermidine	0.402	0.57	763.73+/-226.34	678.11+/-366.49	645.69+/-77.19	633.36+/-85.15	456.02+/-83.92	772.43+/-195.74
Spermine	0.79	0.404	5340+/-332	4129.44+/-1795.21	3831.01+/-1068.73	4462.46+/-1001.2	3455.31+/-1269.53	4429.62+/-895.35
Threonine	0.452	0.588	331.15+/-100.65	235.49+/-158.59	285.71+/-58.46	411.91+/-79.12	271.09+/-198.65	361.75+/-79.09
